# Multi-functional chitosan copolymer modified nanocrystals as oral andrographolide delivery systems for enhanced bioavailability and anti-inflammatory efficacy

**DOI:** 10.1080/10717544.2022.2149894

**Published:** 2022-11-29

**Authors:** Wan Liu, Meng Cheng, Zhiyang Lu, Haocheng Li, Yulin Feng, Yi Jin, Shilin Yang, Jianfang Feng, Liangxing Tu

**Affiliations:** a National Pharmaceutical Engineering Center for Solid Preparation in Chinese Herbal Medicine, Jiangxi University of Chinese Medicine, Nanchang, China; b The Affiliated Hospital, Jiangxi University of Chinese Medicine, Nanchang, China; c School of Pharmacy, Guangxi University of Chinese Medicine, Nanning, China

**Keywords:** Andrographolide, chitosan, bioavailability, real-time distribution, anti-inflammatory

## Abstract

Modifying nanocrystals with functional materials have been common strategy to enlarge the enhancing ability on oral absorption via nanocrystals; however, whether the functional materials have played their full enhancing ability in oral absorption is still unknown. In this study, we synthetized a novel chitosan-based copolymer (the copolymer of sodium dodecyl sulfate (SDS), chitosan (CS) and D-α-Tocopherol polyethylene glycol 1000 succinate, SDS-CS-TPGS), and modified nanocrystals with this copolymer, aiming to enhance the oral absorption of polymer andrographolide (ADR). In real-time distribution study, we found the distribution of ADR, SDS, CS and TPGS varies in gastrointestinal tract, while the distribution of ADR and SDS-CS-TPGS was similar, revealing the SDS-CS-TPGS could able to participate in the absorption process of andrographolide timely. To explore the oral absorption enhancing ability of SDS-CS-TPGS, we prepared a series of nanocrystals modified with different materials and explored their pharmacokinetic performances on SD rats. The results showed the nanocrystals modified with SDS-CS-TPGS (S-C-TANs) exhibited the highest bioavailability, which could enhance the AUC_0-∞_ of ADR from 1.291 mg/L*h to 5.275 mg/L*h (enhanced for about 4.09-folds). The enhanced anti- inflammatory efficacy was also found on ICR mice by employing ear swelling rate, TNF-α, IL-1β and IL-6 and pharmacodynamic index. These results indicated that modified with synthesized copolymer containing different functional stabilizers is an efficient strategy to enlarge the enhancing ability on oral absorption of nanocrystals.

## 1. Introduction

Inflammation and inflammation related diseases are threatening the health of human beings (He et al., 2020; Methenitis et al., 2021), and andrographolide, one of the major diterpenoid extracted from *ANDROGRAPHIS PANICULATA*, is widely used as an anti-inflammatory reagent in China (Tran et al., 2020; Magar et al., 2023). The modern pharmacological studies showed andrographolide has a variety of pharmacological activities, for example anti-inflammatory (Hussain et al., 2022), anti-cancer (Khan et al., 2018), anti-oxidative effects (Wang et al., 2021), anti-thrombosis (Li et al., 2021), inhibiting virus (Latif & Wang, 2020), anti-asthma (Lim et al., 2021), and anti-bacteria (Zhang et al., 2021). However, the clinical application of andrographolide is limited because of its poor water solubility and low bioavailability (Casamonti et al., 2019), and how to improve the oral bioavailability of poor soluble drugs like andrographolide has become a research focus in pharmaceutical field (Zafar et al., 2021).

Nano delivery system has been a popular route to enhance the bioavailability of poor soluble drugs (Marta et al., 2019). To date, researchers have employed multiple nanotechnologies to enhance the oral bioavailability of andrographolide, such as nanoemulsion (Zou et al., 2022), nanoparticles (Chellampillai & Pawar 2011; Parveen et al., 2014), micelles (Zhang et al., 2014), and nanocrystals (Basu et al., 2020). Among these nanotechnologies, nanocrystal technology is more popular due to its advantages like high drug loading, easy preparation and no special requirement for physical-chemical properties of drugs (Zhao et al., 2020; Cheng et al., 2020). In general, nanocrystals enhance the oral absorption of drugs mainly by increasing their saturation solubilities and dissolution rates caused by the decreased particle sizes of nanocrystals (Kesisoglou et al., 2007; Tu et al., 2020). Nanocrystal technology has shown its huge success on oral formulation as there are nearly ten products based on nanocrystals approved by FDA (Lu et al., 2015; Liu et al., 2020; Liu et al., 2021), however, we should bear in mind, that since 2005, no oral nanocrystals was approved. This situation mainly caused by the limited bioavailability enhancing ability of nanocrystals as several researchers found that the bioavailability of some drugs could only be enhanced for less than 2-folds via nanocrystals (Liversidge & Conzentino, 1995; Mou et al., 2011). So, how to enlarge the oral bioavailability enhancing ability of nanocrystals has become a hot research field in nearly years. In recent years, researchers found that with the aid of functional polymer, the oral absorption of nanocrystals could be enhanced (Frank et al., 2015; Haeri et al., 2018). Excluding the enhancing ability on solubility and dissolution rate of nanocrystals, the bioavailability of polymer modified nanocrystals can further improved by enhanced endocytosis-mediated transport, paracellular pathways-introduced transport, M cell-mediated pathway-depended transport, inhibition of P-gp efflux and so on (Mohammad et al., 2019; Mu et al., 2010; Song et al., 2022; Liu et al., 2018). Thus, developing multifunctional polymer modified nanocrystals may be a promising way for further bioavailability enhancement of poorly soluble drugs.

Many researchers have used either physical mixed multi-functional materials or synthesized copolymer to enlarge the oral absorption enhancing ability of nanocrystals (Ahuja et al., 2015; Qiao et al., 2017; Yi et al., 2015), however, whether the functional materials have played their full enhancing ability in oral absorption, or whether physical mixture of multiple functional materials and copolymer insisted with these materials play the same role on oral absorption enhancement is still unknown. To my best knowledge, due to the different physicochemical properties of nanocrystals and functional materials, the drug nanocrystals modified by a single polymer or a physical mixture of multiple polymers made separated in gastrointestinal tract and cannot reach the same site of gastrointestinal tract at the same time, hence limited the absorption enhancing ability of functional materials.

In this study, we intend to study the real-time distribution of drug and modified materials in gastrointestinal tract by labeling these materials with different fluorescent probes, and operated on a small animal live imaging system. The model functionals materials we selected were: (1) sodium dodecyl sulfate, which could open the tight junction between cells (Bezrodnykhet al., 2021), (2) chitosan, which could increase contact time with mucous membranes via its bioadhesive behavior (Zoya et al., 2021; Chen et al., 2020; Liu et al., 2020), (3) D-α-Tocopherol polyethylene glycol 1000 succinate, a P-gp efflux inhibitor (Chen et al., 2021; Rathod et al., 2021).

## 2. Materials and methods

### 2.1. Materials

Sodium dodecyl sulfate (SDS), sodium chloride, ethyl acetate, dichloromethane and methanol were purchased from Xilong Scientific Chemical Reagent Co., Ltd, China. D-α-Tocopherol polyethylene glycol 1000 succinate (TPGS), chitosan (CS), hydrochloric acid (HCl), triethylamine (TEA), succinic anhydride (SA) and 1-Ethyl-3-(3-dimethylaminopropyl) carbodiimide hydrochloride (EDC·HCl) was purchased from Aladdin, China. 4-dimethylaminopyridine (DMAP) was purchased from Energy Chemical, China. Andrographolide (purity > 98%) was purchased from Shanxi Tengmai Biotechnology co., Ltd, China. N-Hydroxysul fosuccinimide sodium salt (NHS) and 2-morpholinoethanesulfonic acid were purchased from Macklin, China. Arachidonic acid was purchased from Shanghai Kaiwei Chemical Technology Co., Ltd, China. Indomethacin was purchased from Shanghai Macklin Biochemical Technology Co., Ltd, China. Mouse TNF-α ELISA kit, mouse IL-1β ELISA kit and mouse IL-6 ELISA kit were purchased from Shanghai Jianglai Biotechnology Co., Ltd, China. HPLC-grade methanol was used for high performance liquid chromatography (HPLC). Acridine orange and flourescein isothiocyante isomer were purchased from Rhawn, China. Rhodamine B was purchased from Solarbio, China. All other materials and reagents were of analytical grade and purified water was used throughout this study.

Male Sprague Dawley (SD) rats, weighing 240-260 g and male mouse, weighing 25-30 g were supplied by Hunan SJA Laboratory Animal Co., Ltd, China.

### 2.2. Synthesis and characterization of SDS-CS-TPGS copolymer

**Figure 1.**
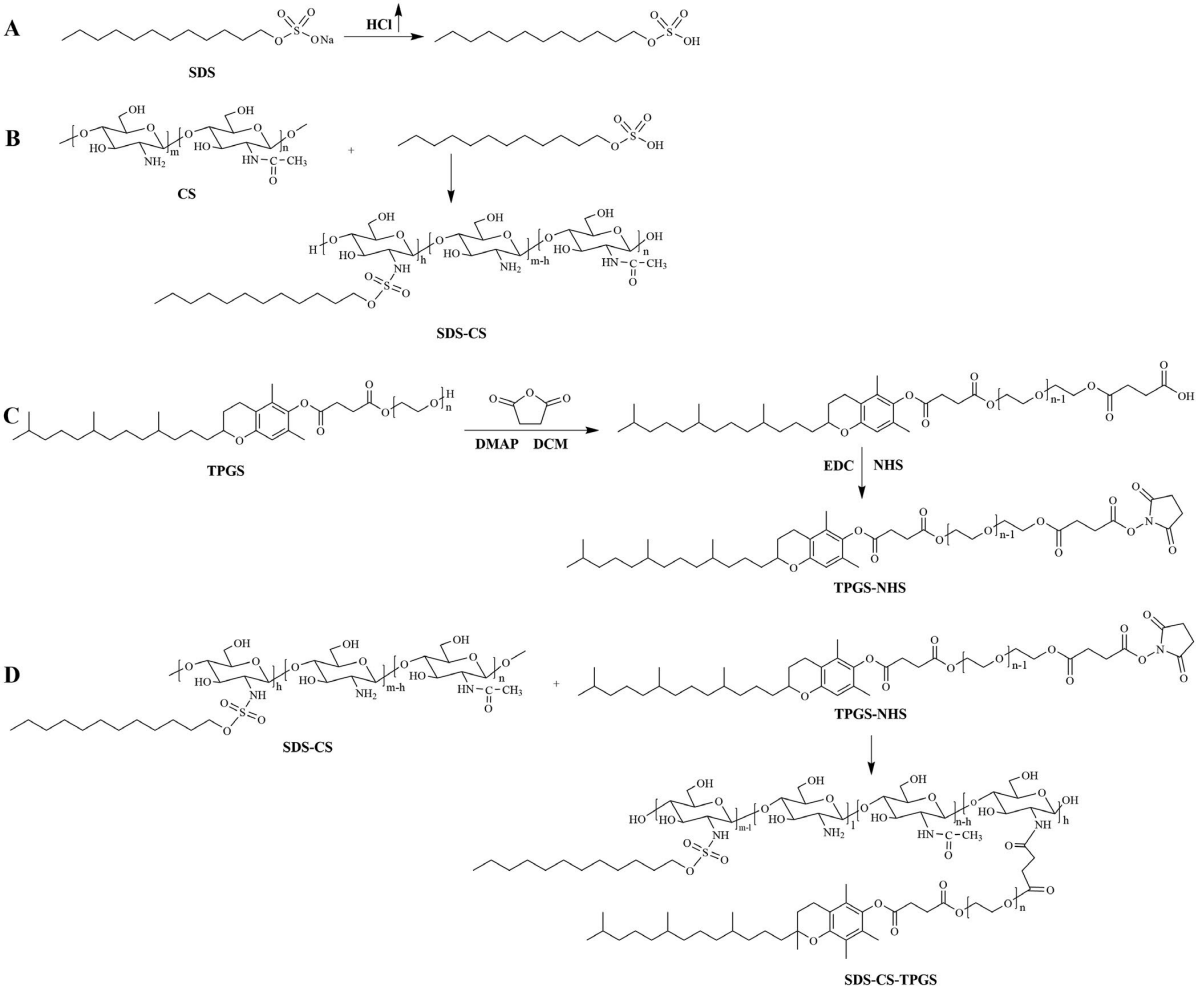
The synthesis process (A) of the SDS-CS-TPGS copolymer.

The synthesis of SDS-CS-TPGS copolymer included four primary steps (Figure 1): (1) SDS acidification reaction: Adding SDS to methanol, and adding enough hydrochloric acid gas until the SDS methanol solution become clear, and then the SDS acidifier was obtained after removing methanol by rotary evaporation; (2) The synthesis of SDS-CS polymer: First, weighing 3.4 g CS and add it into 250 mL round-bottled flask, and then 100 mL water and 20 mL hydrochloric acid solution (5.0 mL→100 mL) were added to dissolve CS. Second, mixing 7 mL 0.23 mM dimethyl aminopyridine ethanol solution and 7 mL 0.27 mM acidified SDS aqueous solution, and then reacted at 80 °C for 24 hours under the protection of nitrogen. Finally, the reaction product was centrifuged after cooled it to room temperature, and the supernatant was dialyzed by water and 50% ethanol solution for 4 h in triple and the SDS-CS was obtained by rotary evaporation and freeze-drying, thereafter; (3) The activation of TPGS (Chen et al., 2018): Weighing a certain amount of TPGS and succinic anhydride with molar ratio of 1:1.2, and mixed them with DMAP in a round-bottomed flask. After drying in a vacuum drying oven for 4 hours, these materials were dissolved by purified dichloromethane, and then reaction were operated by adding anhydrous triethylamine, and heating to reflux and stirring in an oil bath for 24 hours under nitrogen protection, and the viscous liquid TPGS succinate monoester was gained by purified with chloromethane-ethyl acetate-methanol solution (the ratio =9:2:2) and silica gel column. The TPGS succinic acid monoester was condensed with EDC·HCl and NHS, briefly: Weighing TPGS-COOH, EDC·HCl, and NHS in a molar ratio of 1: 1.2: 1.2, then dissolving TPGS-COOH and EDC·HCl with morpholine ethanesulfonic acid buffer (pH = 5.0), stirring in an ice bath for 10 min, then reacting at room temperature for 6 hours); (4) The synthesis of SDS-CS-TPGS copolymer: SDS-CS and activated TPGS was dissolved by water, and reacted at room temperature for 24 hours. The SDS-CS-TPGS copolymer was gained after purified with dialysis bag and dried by freeze-dryer. The structure of new synthesized copolymers was confirmed by using ^1^H NMR and FT-IR spectroscopy.

### 2.3. Preparation of the nanocrystals

#### 2.3.1. Preparation of andrographolide nanocrystals (ANs)

Andrographolide nanocrystals (ANs) were prepared by high pressure homogenization. Firstly, andrographolide was dispersed in 50 mL purified water and pretreated by high shear homogenizer at 13000 rpm for 10 min using Fluko® FA25 (FLUKO, Germany). Secondly, the crude suspension was homogenized by high pressure homogenization machine (AH-NANO, ATS, China) with optimized parameters of 50 bar for 2 cycles, 200 bar for 2 cycles, 500 bar for 2 cycles, 1000 bar for 20 cycles, with homogenization temperature of 41-50 °C.

#### 2.3.2. Preparation of stabilizer modified andrographolide nanocrystals (SANs, TANs and S-C-TANs)

Sodium dodecyl sulfate modified andrographolide nanocrystals (SANs) were prepared by high pressure homogenization with optimized parameters. Briefly, 250 mg andrographolide and 70.8 mg SDS were weighed and dispersed in 50 mL purified water. After high shear homogenization at 13, 000 rpm for 10 min using Fluko® FA25, the suspension was homogenized by high pressure homogenizier with homogenization pressure of with optimized parameters of ANs. D-α-Tocopherol polyethylene glycol 1000 succinate modified andrographolide nanocrystals (TANs) was prepared by high pressure homogenization with optimized parameters of ANs. Briefly, 429.2 mg TPGS were weighed and dispersed in 50 mL purified water. After high shear homogenization at 13, 000 rpm for 10 min using Fluko^®^ FA25, the suspension was homogenized by high pressure homogenizier with optimized parameters of ANs. The SDS-CS-TPGS copolymer modified andrographolide nanocrystals (S-C-TANs) was prepared by high pressure homogenization with optimized parameters of ANs. Briefly, 250 mg andrographolide and 500 mg SDS-CS-TPGS were weighed and dispersed in 50 mL purified water. After high shear homogenization at 13,000 rpm for 10 min, the suspension was homogenized with optimized parameters of ANs.

### 2.4. Characterization of nanocrystals

The characterization of ANs, SANs, TANs and S-C-TANs was performed as below:

After the sample concentration was diluted to 0.1 mg/mL (calculated as andrographolide), the particle sizes, polydispersity indices (PDI) and zeta potentials of ANs, SANs, TANs and S-C-TANs were characterized with Nano ZS 90 nanoparticle sizer (Malvern Instruments Co., Ltd., UK), and all samples were analyzed in triplicate.

The morphology of andrographolide was performed on a scanning electron microscope (SEM) (quanta 250, Fei, America), while the morphology of ANs, SANs, TANs and S-C-TANs was analyzed on a transmission electron microscope (TEM) (Tecnai spirit, Fei America).

The crystalline states of ANs, SANs, TANs and S-C-TANs were studied by employing x-ray diffraction (XRD) (D/max 2500 PC, Japan Science Co., Ltd., Japan).

### 2.5. Saturated solubility study

The saturation solubility of ADR was studied in hydrochloric acid aqueous solution (HCl, pH 1.2), citrate - disodium hydrogen phosphate buffer (C-DHPB, pH 4.0), phosphate buffered saline (PBS, pH 6.8) and purified water, respectively, at 37 °C, while the saturation solubility of SDS and ADR physical mixture (SDS + ADR), TPGS and ADR physical mixture (TPGS + ADR) were studied in purified water at 37 °C. Briefly, excess formulation powders were added into 5 mL buffers, and shaked at 37 °C for 24 h, then the undissolved drug was removed by centrifuging at 13, 000 rpm for 10 min and filtering with 0.22 μm microporous membrane. The drug concentration in the filtrate was determined by HPLC. The freshly prepared ANs, SANs, TANs and S-C-TANs were directly shaking for 24 h at 37 °C, and treated as that of andrographolide, thereafter. ADR was analyzed at 225 nm and at 35 °C using HPLC linked to Ultimate^Ⓡ^ XB-C18 (4.6 mm × 250 mm, 5 μm) column. The mobile phase which consisted of methanol and water (60:40, v/v) was pumped at 1 mL/min. The HPLC analysis met the methodological requirements by validating.

### 2.6. Pharmacokinetic study

We explored oral absorption enhancing ability of andrographolide nanocrystals by operating pharmacokinetic studies on SD rat. In brief, twenty-five SD rats were randomly divided into 5 groups, and administrated ADR-water (dispersed ADR in water), ANs, SANs, TANs and S-C-TANs at 50 mg/kg by gavage (Chen et al., 2014). After administrated for 0.083, 0.25, 0.5, 0.75, 1, 2, 4, 6, 8, 12 and 24 h, about 0.3 mL blood sample was collected by retro-orbital puncture. The plasma was centrifuged at 4,000 r/min for 10 min at 4 °C and stored at −20 °C. The andrographolide concentrations in plasma samples were detected after treatments as following: 50 μL plasma samples, 25 μL luteolin solution (internal standard 10.11 μg/mL) and 200 μL methanol solution were mixed by vortexing for 5 min. The mixed solution was subsequently centrifuged at 12,000 rpm for 10 min and the supernatant was collected and dried at room temperature under a stream of nitrogen. The residue was reconstituted with 50 μL methanol and mixed by vortexing for 3 min. after centrifuging at 12,000 rpm for 10 min, the supernatant was injected into the HPLC system for analysis. The ADR concentration in plasma samples was detected by HPLC following the conditions shown in supplementary materials, and pharmacokinetic parameters were calculated using the DAS 3.3.0 pharmacokinetics program (developed by the Clinical Trial Center of Shanghai University of Traditional Chinese Medicine, Shanghai, China).

### 2.7. Real-time distribution of nanocrystals in the gastrointestinal tract

We performed real-time distribution study to explore whether the functional materials have played their full enhancing ability in oral absorption. We used acridine orange to label SDS, fluorescein isothiocyanate isomer I to label TPGS, CS, SDS-TPGS and SDS-CS-TPGS, and rhodamine B to label ADR, respectively. After labeling the samples, seventy-two SD rats were randomly divided into 6 groups, named SDS group, TPGS group, ADR group, CS group, SDS-CS-TPGS group and SDS-CS-TPGS + ADR group. The SDS-CS-TPGS + ADR group was administered with SDS-CS-TPGS and ADR at the same time. The animals were killed by cervical dislocation at the pre-set time points of 0.5 h, 1 h, 2 h and 3 h. Then the gastrointestinal tract was removed in time and put it into a small animal live imaging system (Lumina XR, PerkinElmer, America) for photographic observation.

### 2.8. Study on anti-inflammatory effect of S-C-TANs

To explore whether the enhancing on bioavailability could result in enhanced therapeutic effect, we studied the anti-inflammatory efficacy of nanocrystal. The ear swelling ICR mice model was established by using arachidonic acid and the anti-inflammatory efficacy was evaluated by employing ear swelling rate of ICR mice serum levels of TNF-α, IL-6 and IL-1β, as pharmacodynamic index. In this study, fifty-six ICR mice were randomly divided into 8 groups, named control group (normal animal), model group (without drug), indomethacin group (positive drug, 10 mg/kg, po), ADR injection group (10 mg/kg, iv), ADR group (100 mg/kg, po), L-S-C-TANs group (low dose of S-C-TANs, 25 mg/kg, po), M-S-C-TANs group (middle dose of S-C-TANs, 50 mg/kg, po) and H-S-C-TANs group (high dose of S-C-TANs, 100 mg/kg, po). After treated with different drugs for 4 days, 25 μL arachidonic acid solution (200 mg/mL) was smeared on the right ear of ICR mice, and after administrated for 0.5 h, the serum was taken, and stored at −20 °C for detection of TNF-α, IL-1β and IL-6. Ears were cut off along the auricle baseline, and samples were taken from the same part of the left and right ears of the mice with an ear punch with diameter of 8 mm. After the samples were taken and weighed, the swelling degree was indicated by the weight difference between the left and right ears. Cellular inflammatory factors, like TNF-α, IL-1β, and IL-6, were measured by Elisa Kit.

### 2.9. Statistical analysis

All values were expressed as the means ± SD. The statistical analysis was performed using one-way ANOVA with SPSS Statistics 22.0 (SPSS Inc., Chicago, America). The differences were considered significant at *p* < 0.05.

## 3. Results and discussion

### 3.1. Synthesis of SDS-TPGS copolymer

**Figure 2.**
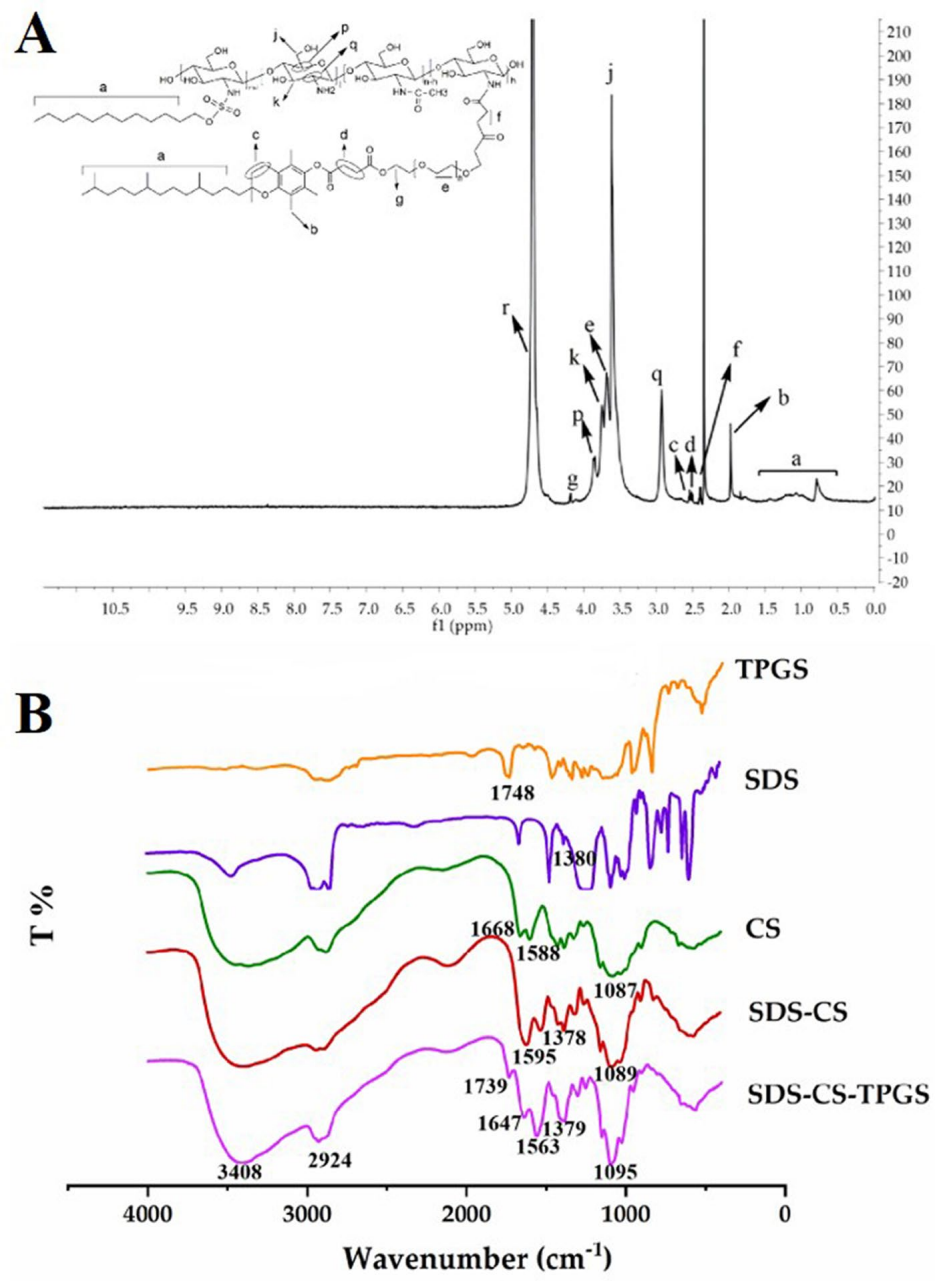
The 1H NMR spectra (B) and FT-IR spectra (C) of the SDS-CS-TPGS copolymer.

The synthesized SDS-CS-TPGS copolymer was identified by ^1^H NMR and FI-TR. As shown in Figure 2A, the structure of SDS-CS-TPGS contains the hydrogen peaks of ‘a’ (δ0.7 7 3 ∼ 1.432 ppm), ‘b’ (δ1.970 ppm), ‘c’ quartet (δ2.520 ppm, 2.526 ppm, 2.537, 2.549 ppm), ‘d’ (δ 2.927 ppm), ‘f’ (quartet peak, δ 2.374 ppm, 2.385 ppm, 2.396 ppm, 2.410 ppm), ‘q’ (δ 2.927 ppm), ‘j’ (doublet peak, δ 3.606, 3.619 ppm), ‘e’ (doublet peak, δ 3.669, 3.687 ppm), ‘k’ (doublet peak, δ 3.734, 3.752 ppm), ‘p’ (doublet peak, δ 3.847, 3.868 ppm), ‘g’ (quartet peak, δ4.177 ppm, 4.182 ppm, 4.184 ppm, 4.192 ppm), ‘r’ (solvent peak, δ4.701 ppm), and these peaks could belong to SDS, CS or TPGS, indicating the ^1^H NMR spectrum was consistent with the theoretical ^1^H NMR spectrum of SDS-CS-TPGS.

Then, we confirmed the results gained from ^1^H NMR by investigating the FI-TR profiles of different materials. Briefly, dry potassium bromide was mixed with SDS, TPGS, CS, SDS-CS and SDS-CS-TPGS, respectively, in a suitable proportion, and then the samples were pressed and tested by FI-TR to obtain the infrared spectrograms (Figure 2B). The special infrared peak of TPGS and SDS was ‘C = O’ (1748 cm^−1^) and ‘S = O’ (1380 cm^−1^), respectively, and CS exhibited special infrared peaks like ‘C = O’ (1588 cm^−1^), ‘N-H’ (1668 cm^−1^) and ‘C-O-C’ (1087 cm^−1^). In the spectra of SDS-CS, the ‘C = O’ (1595 cm^−1^), ‘S = O’ (1378 cm^−1^) and ‘C-O-C’ (1089 cm^−1^) existed, while the ‘N-H’ (1668 cm^−1^) was missing, indicating the acidified SDS could react with CS to form SDS-CS. The special infrared peaks in the SDS-CS-TPGS were ‘C = O’ (1739 cm^−1^) ‘C = O’ (1647 cm^−1^) ‘N-H’ (1563 cm^−1^) ‘S = O’ (1379 cm^−1^) ‘C-O-C’ (1095 cm^−1^) and ‘C = O’ (1739 cm^−1^), indicating that TPGS was connected to the SDS-CS, and the SDS-CS-TPGS copolymer was successfully synthesized.

### 3.2. Characterization of nanocrystals

In order to explore the oral absorption enhancing ability of SDS-CS-TPGS copolymer and nanocrystals, we prepared several kinds of nanocrystals: stabilizer-free andrographolide nanocrystals (ANs), SDS modified andrographolide nanocrystals (SANs), TPGS modified andrographolide nanocrystals (TANs) and SDS-CS-TPGS modified andrographolide nanocrystals (S-C-TANs), by high-pressure homogenization, and the production process was provided in supplementary materials. Then these nanocrystals were characterized by Nano ZS 90 nanoparticle sizer (Malvern Instruments Co., Ltd., UK), transmission electron microscopy (TEM (Tecnai spirit, Fei America)), X-ray diffraction (XRD (D/max 2500 PC, Japan Science Co., Ltd., Japan), etc.

**Figure 3.**
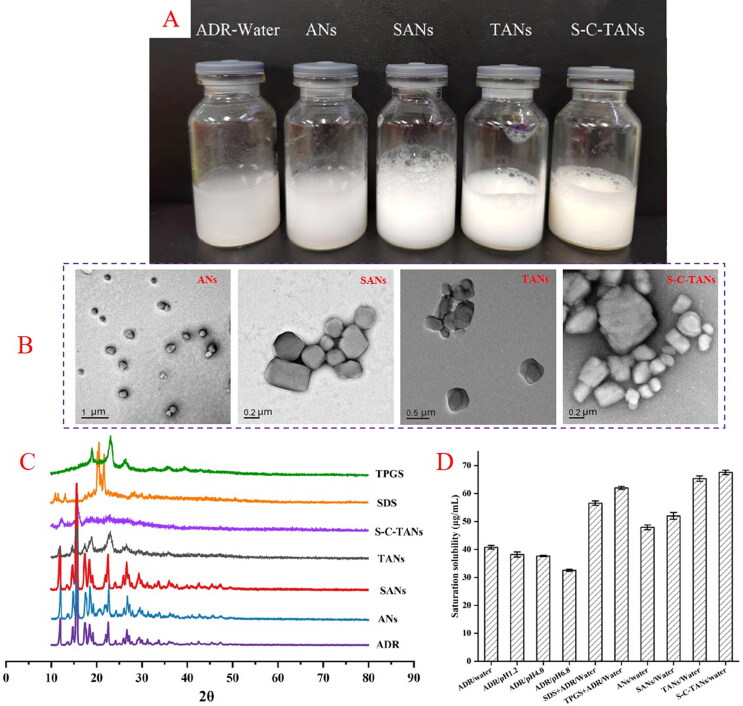
The appearance (A), TEM paragraph (B), XRD spectra (C) and saturation solubility (D) of andrographolide nanocrystals.

The appearance of fresh prepared ADR-Water, ANs, SANs, TANs and S-C-TANs were shown in Figure 3(A), ANs, SANs, TANs and S-C-TANs were milky white suspension, while ADR-Water that has obvious large particles and was easy to settle and stratify. The particle sizes of ANs, SANs, TANs and S-C-TANs were 368.3 ± 4.7 nm, 506.1 ± 8.1 nm, 552.5 ± 4.9 nm and 573.2 ± 3.9 nm, respectively. That PDI values were above 0.3 indicate high heterogeneity, while the PDI of ANs, SANs, TANs and S-C-TANs were 0.207 ± 0.021, 0.191 ± 0.017, 0.106 ± 0.089 and 0.223 ± 0.024. PDI values of those nanocrystals were all below 0.3, indicating the distribution of these nanocrystals were relatively uniform (Imam et al., 2015). It could be seen from the TEM spectra that the appearance of ANs liked a melon seed shell, while the SANs, TANs, and S-C-TANs were irregular spherical-liked (Figure 3B).

The crystalline states of ADR, SDS, TPGS, ANs, SANs, TANs and S-C-TANs were studied by employing x-ray diffraction (XRD) (Ultima IV, Rigaku, Tokyo, Japan). The XRD patterns of ADR, SDS, TPGS, ANs, SANs, TANs and S-C-TANs were depicted in Figure 3(C). The characteristic peaks of ADR presented at 2θ = 11.91°, 14.68°, 15.44°, 17.46°, 18.44°, 19.22°, 22.61°, 24.40°, 26.68°, 27.26°, 29.54°, 31.16°, 33.77°, 36.06°, 40.90°, 42.48°, and the characteristic peaks of SDS presented at 2θ = 11.06°, 11.66°, 13.32°, 20.62°, 21.82°, while the characteristic peaks of TPGS presented at 2θ = 19.06°, 23.26°, 26.26°. It could be seen from the paragraphs that the diffraction peaks of ANs and SANs are roughly the same as those of ADR, indicating the crystalline form of nanocrystals has not changed under high pressure homogenization or modified by SDS. The characteristic peaks of TANs presented at 2θ = 11.91°, 15.44°, 17.46°, 19.12°, 23.26°, and 26.46°, and the diffraction peaks became less and weaker, showing the TANs was partly transformed from crystal state to amorphous state. excluding the peaks at 2θ were 12.31° and 15.88°, there was no other diffraction peak in the XRD spectra of S-C-TANs, indicating the nanocrystals were nearly in amorphous.

### 3.3. The saturation solubility of andrographolide and nanocrystals

Figure 3(D) exhibited the saturation solubilities of ADR and its nanocrystals, and the results showed with the increasing of pH, the saturation solubility of ADR decreased. The saturation solubility of ADR in water at 37 °C was 40.79 ± 0.64 μg/mL, and after transformed the ADR into nanocrystals, the solubility was slightly increased for about 1.17-folds (shown in Table S1). The saturated solubility of nanocrystals was further increased after being modified by functional materials.

### 3.4. Pharmacokinetic study

**Figure 4.**
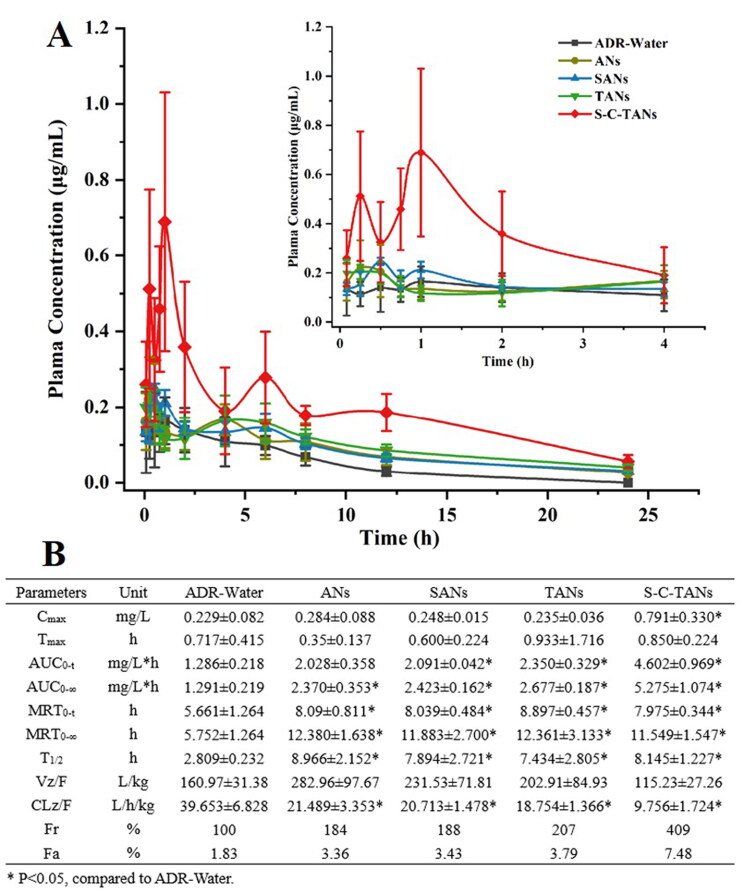
The pharmacokinetic curves (A) and parameters (B) andrographolide nanocrystals (mean ± SD, *n* = 5).

We explored oral absorption enhancing ability of andrographolide nanocrystals by operating pharmacokinetic studies on SD rat. The pharmacokinetic behaviors of ADR, ANs, SANs, TANs and S-C-TANs are shown in Figure 4. Compared with the ADR-Water group, the AUC_0-∞_ of the ANs increased from 1.291 mg/L*h to 2.370 mg/L*h, indicating nanocrystals could significantly increase the oral absorption of andrographolide. After modified with SDS or TPGS, no significant change happened on C_max_ of nanocrystals, while the AUC_0-∞_ was slightly increased. After modified with SDS-CS-TPGS copolymer, nanocrystals gained the highest Cmax and bioavailability. The C_max_ was enhanced from 0.284 ± 0.088 μg/mL for ANs to 0.791 ± 0.330 μg/mL for S-C-TANs. Compared with ADR-Water, the relative bioavailability of ANs, SANs, TANs, and S-C-TANs were 184%, 188%, 207%, 240%, 297%, and 409%, respectively, and the absolute bioavailability of andrographolide (ADR) and S-C-TANs was 1.83% and 7.48%, respectively. The pharmacokinetic study of ADR-Water and ANs showed that nanocrystal technology could significantly improve the oral bioavailability of ADR, and the comparison of the pharmacokinetic parameters of SANs, TANs and ANs showed that the modification of drug nanocrystals by functional materials can further improve the oral bioavailability of andrographolide. Among these functional materials, the SDS-CS-TPGS copolymer, formed by chemical synthesis of different functional materials showed the highest ability on enhancing oral bioavailability of andrographolide. The first peak in pharmacokinetic curves is caused by the absorption of drug, while the peak emerged at about 6 h is caused by the enterohepatic circulation (Guan & Morris 2019).

### 3.5. Real-time distribution in the gastrointestinal tract

**Figure 5.**
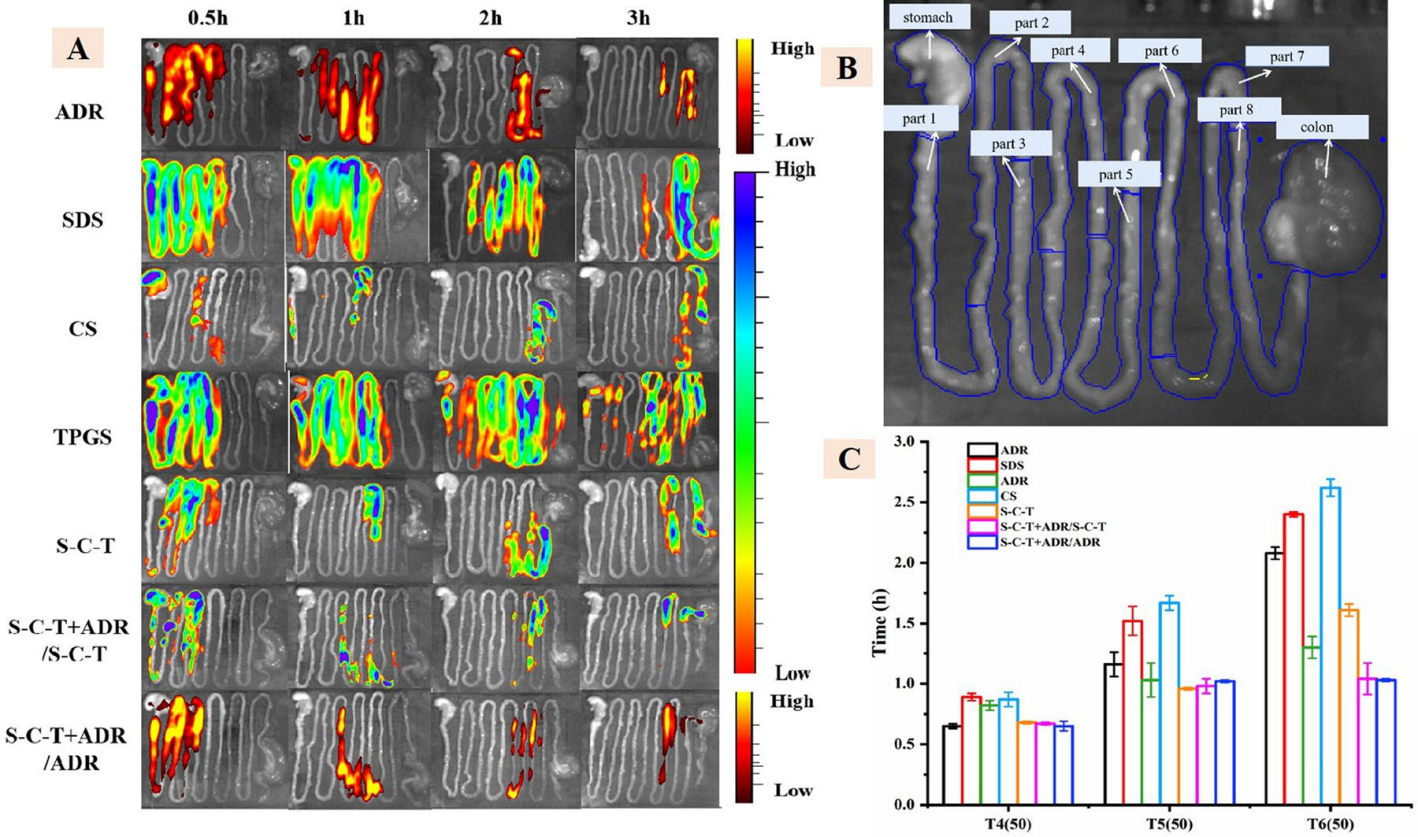
The real-time distribution (A), gastrointestinal segments (B) and passing time(C) of 50% materials through the fourth, fifth and sixth segments of small intestine (mean ± SD, *n* = 3).

The distribution of gastrointestinal segments in each group of animals at each time period was observed by in live imaging system (Figure 5A). We could know that the fluorescence intensity of each group decreases as time went on, and we could preliminarily see that the distribution of SDS, CS and TPGS in the gastrointestinal tract of SD rats was different from the distribution of ADR, while the distribution of SDS-CS-TPGS in the gastrointestinal tract is roughly the same as the distribution of ADR, when SDS-CS-TPGS and ADR were administered at the same time. In order to further understand the distribution of various materials and ADR in the gastrointestinal tract, we divided the gastrointestinal tract of SD rats into ten parts such as stomach, small intestine which divided into 8 sections on average and colon, as shown in Figure 5(B). The Figure 5(C) and Table S2 (in supplementary materials) showed the fluorescence intensity statistics of the distribution of polymer materials and ADR in various parts of the gastrointestinal tract, which can more intuitively reflect that the time of the site was close that they reached the same level in the gastrointestinal tract of SD rats when SDS-CS-TPGS and ADR are administered at the same time. For example, when SDS-CS-TPGS were taken orally with ADR, the time of 50% SDS-CS-TPGS passing part 4, part 5 and part 6 of small intestine was 0.67 ± 0.01 h, 0.98 ± 0.06 h and 1.04 ± 0.13 h, respectively, which were not much different from that of ADR, as the passing time of ADR in part 4, part 5 and part 6 was 0.65 ± 0.04 h, 1.02 ± 0.01 h and 1.03 ± 0.01 h, respectively. However, these times of other materials and ADR were different, indicating the materials and ADR could not reach the same site of gastrointestinal tract at the same time, which may limit the absorption enhancing ability of functional materials. Combining the results described above, we can conclude that materials in physical mixture may separate in intestinal, and synthesized copolymer could reduce the separation and has better ability to research the absorption site of drug at the same time.

### 3.6. The anti-inflammatory effect of S-C-TANs

To explore whether the enhancing on bioavailability could result in enhanced therapeutic effect, we studied the anti-inflammatory efficacy of nanocrystal. The ear swelling ICR mice model was established by using arachidonic acid and the anti-inflammatory efficacy was evaluated by employing ear swelling rate of ICR mice serum levels of TNF-α, IL-6 and IL-1β, as pharmacodynamic index. In this study, fifty-six ICR mice were randomly divided into 8 groups, named control group (normal animal), model group (without drug), indomethacin group (positive drug, 10 mg/kg, po), ADR injection group (10 mg/kg, iv), ADR group (100 mg/kg, po), L-S-C-TANs group (low dose of S-C-TANs, 25 mg/kg, po), M-S-C-TANs group (middle dose of S-C-TANs, 50 mg/kg, po) and H-S-C-TANs group (high dose of S-C-TANs, 100 mg/kg, po). After treated with different drugs for 4 days, 25 μL arachidonic acid solution (200 mg/mL) was smeared on the right ear of ICR mice, and the ears and blood samples were harvested after 0.5 h.

**Figure 6.**
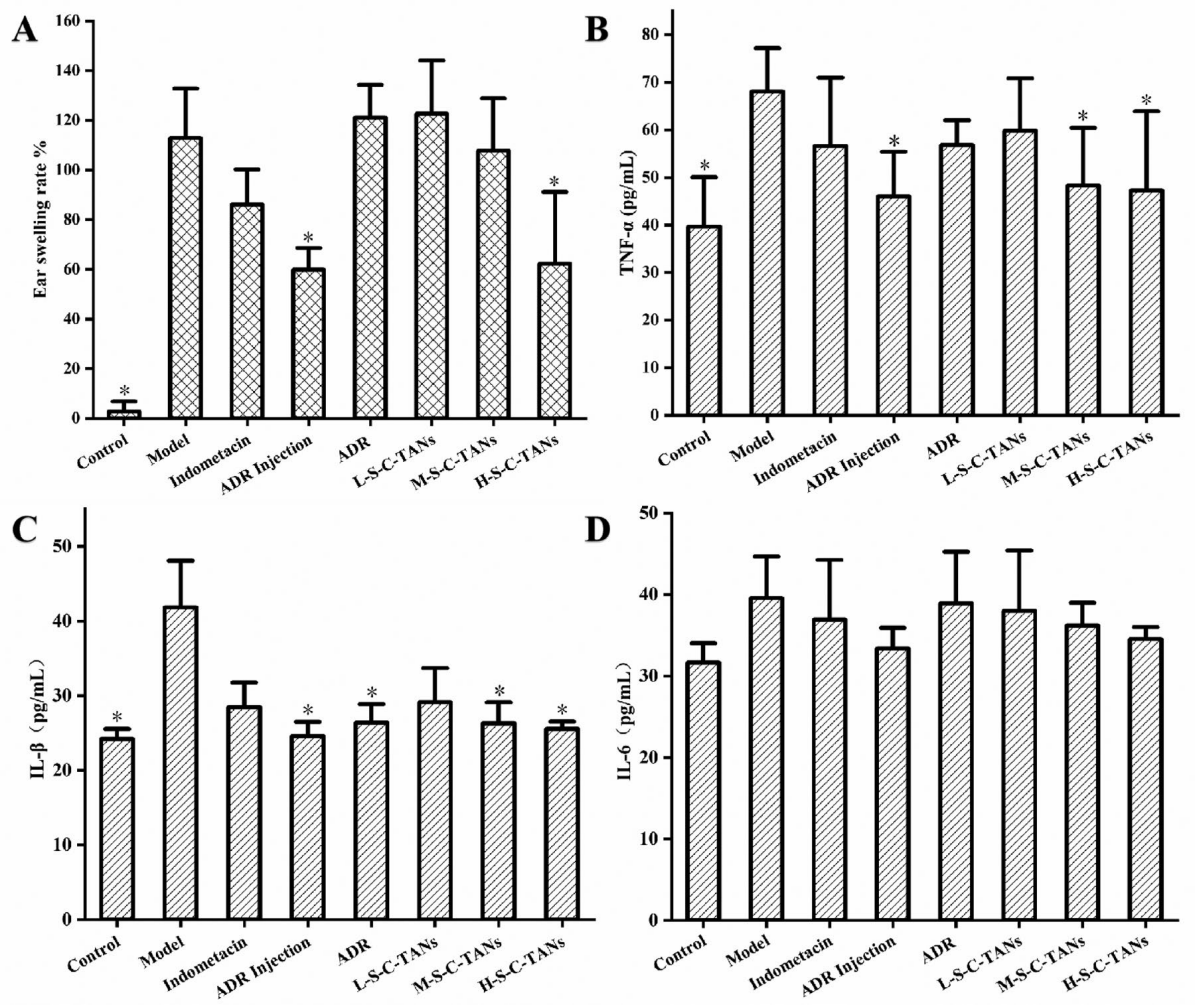
The effect of nanocrystals on ear swelling rate (A), TNF-α (B), IL-1β (C) and IL-6 (D) in mice (mean ± SD, *n* = 7, *P < 0.05 vs. model group).

The ear swelling ICR mice model was successfully established as there were significant difference on pharmacodynamic index between model group and control group (Figure 6). Comparing the treatment groups, we could find that the ear swelling rate of the mice in the injection group and the H-S-C-TANs group decreased significantly (Figure 6A). The anti-ear swelling efficacy of all groups were as follow: ADR injection group > H-S-C-TANs group > positive drug group > M-S-C-TANs group > ADR group ≈ L-S-C-TANs group. The effects of nanocrystals on cellular inflammatory factors TNF-α, IL-1β and IL-6 in ear swelling mice were shown in Figure 6(B)-D. Compared with the control group, the values of TNF-α and IL-1β in the model group changed significantly, indicating the model stimulated TNF-α and IL-1β in mice, while the IL-6 factor showed no significant difference between the all groups, indicating the model did not stimulate the changes of IL-6 cell inflammatory factors in mice. From the results of TNF-α and IL-1β studies, there were significant differences between the model group and the ADR injection group, M-S-C-TANs group and H-S-C-TANs group, and the TNF-α and IL-1β levels of H-S-C-TANs group were similar to that of ADR injection group. The above results indicated the enhanced bioavailability could lead to enhanced pharmacodynamic effect, and SDS-CS-TPGS modified nanocrystals (100 mg/kg) have the similar anti-inflammatory efficacy with ADR injection (10 mg/kg).

## 4. Conclusion

In this study, we employed three different functional stabilizers to synthesize multifunctional SDS-CS-TPGS copolymer and used this copolymer to modify the andrographolide nanocrystals (S-C-TANs). The particle size and zeta potential of S-C-TANs was 573.2 ± 3.9 nm and 46.27 ± 0.25 mV, and the saturation solubility of andrographolide was increased for about 1.7-folds by nanocrystals. With the aids of increased solubility and absorption enhancing efficacy of SDS-CS-TPGS, the C_max_ and AUC_0-∞_ of andrographolide was enhanced from 0.229 ± 0.082 mg/L, 1.291 ± 0.219 mg/L*h to 0.791 ± 0.330 mg/L and 5.275 ± 1.074 mg/L*h, respectively. In addition, we confirmed the synthesized SDS-CS-TPGS copolymer could increase the absorption enhancing potential of multiple functional materials by maintaining the similar distribution behaviors of drug and materials in gastrointestinal tract. The enhanced bioavailability leads to enhanced anti- inflammatory efficacy of nanocrystals by performing on arachidonic acid induced inflammation ICR mice model. This study indicated that modified with synthesized copolymer containing different functional stabilizers is an efficient strategy to enlarge the enhancing ability on oral absorption of nanocrystals.

## Supplementary Material

Supplemental Material

## Data Availability

The data that support the findings of this study are available from the corresponding author, L. Tu, upon reasonable request.
