# Supplementary material for: Multi-functional chitosan copolymer modified nanocrystals as oral andrographolide delivery systems for enhanced bioavailability and anti-inflammatory efficacy
Source: Drug Deliv. 2022 Nov 29;29(1):3432–42. doi: 10.1080/10717544.2022.2149894 (PMC9718561; doi:10.1080/10717544.2022.2149894)
Supplement: Supplemental Material [file IDRD_A_2149894_SM5939.docx]

**Multi-functional chitosan copolymer modified nanocrystals as oral andrographolide delivery systems for enhanced bioavailability and anti-inflammatory efficacy**

Wan Liu ^a,1^, Meng Cheng ^a,c,1^, Zhiyang Lu ^a^ , Haocheng Li ^a^, Yulin Feng ^a^, Yi Jin ^a^, Shilin Yang ^a^, Jianfang Feng ^a,b,^*, Liangxing Tu ^a,*^

**SUPPLEMENTARY MATERIALS:**

**1 Preparation of stabilizer modified andrographolide nanocrystals**

ANs, SANs, TANs and S-C-TANs was prepared by high pressure homogenization. Briefly, ANs (250 mg andrographolide), SANs (250 mg andrographolide and 70.8 mg SDS), TANs (250 mg andrographolide and 429.2 mg TPGS) and S-C-TANs (250 mg andrographolide and 500 mg SDS-CS-TPGS) were dispersed in 50 mL purified water. After high shear homogenization at 13, 000 rpm for 10 min using Fluko® FA25 (FLUKO, Germany), the suspension was homogenized by high pressure homogenizers (AH-NANO, ATS, China) with homogenization pressure of 50 bar for 2 cycles, 200 bar for 2 cycles, 500 bar for 2 cycles and 1000 bar for 20 cycles, with homogenization temperature of 41-50℃.

**2 The saturation solubility and log P of andrographolide and nanocrystals**

The saturation solubility of ADR powder was studied in purified water, hydrochloric acid aqueous solution (HCl, pH 1.2), citrate - disodium hydrogen phosphate buffer (C-DHPB, pH 4.0) and phosphate buffered saline (PBS, pH 6.8), respectively, at 37 ℃, while the saturation solubility of ADR and SDS physical mixture (SDS+ADR), ADR and TPGS physical mixture (TPGS+ADR), ANs, SANs, TANs and S-C-TANs were studied in purified water at 37 ℃. Briefly, excess formulation powders were added into 5 mL buffers, and sharked at 37℃ for 24 h, then the undissolved drug was removed by centrifugation at 13, 000 rpm for 10 min and filtration with 0.22 μm microporous membrane. The drug concentration in the filtrate was determined by HPLC. The fresh prepared STANs and S-TANs were directly shaking for 24 h at 37 ℃, and treated as that of andrographolide, thereafter. ADR was analyzed at 225 nm using HPLC linked to UltimateⓇ XB-C18 (4.6 mm × 250 mm, 5μm) column. The mobile phase, consisting of methanol and water (60:40, v/v), was pumped at 1 mL/min. The column temperature was 35℃. The HPLC analysis was validated and met the methodological requirements.

The oil-water partition coefficient (log P) of ADR was studied in purified water, pH 1.2, pH 4.0 and pH 6.8 at 25℃ and 37 ℃. ANs, SANs, TANs and S-C-TANs were studied in purified water at 25℃ and 37 ℃, respectively. Briefly, 4 mL water or water solution (e.g. purified water, pH 1.2, pH 4.0 and pH 6.8) was used to dissolve different formulations, and then 4 mL octyl alcohol was added in these tubes, respectively. The solutions were operated at 25 ℃ or 37 ℃ for 24 h, and then the drug concentration in the two phases was determined by HPLC.

Combining the saturated solubility and log P of each group (shown in Table S1, it could be seen that the greater the saturated solubility is, the greater the log P value is.

**Table S1**

The saturation solubility of andrographolide and nanocrystals and the oil-water partition coefficient (Log P) of andrographolide and nanocrystals (Mean±SD, n=3).

| Sample | Saturation solubility μg/mL | Log P | |
| --- | --- | --- | --- |
|  | 37℃ | 25℃ | 37℃ |
| ADR/Water | 40.79±0.64 | 1.78±0.02 | 1.76±0.01 |
| ADR/pH1.2 | 38.21±0.95 | 1.85±0.02 | 1.84±0.02 |
| ADR/pH4.0 | 37.68±0.24 | 1.85±0.02 | 1.87±0.02 |
| ADR/pH6.8 | 32.53±0.42 | 1.88±0.02 | 1.94±0.02 |
| SDS+ADR/Water | 86.56±0.26 | 1.72±0.01 | 1.78±0.01 |
| TPGS+ADR/Water | 72.45±1.32 | 1.41±0.02 | 1.53±0.01 |
| ANs/Water | 47.91±0.85 | 1.71±0.02 | 1.84±0.03 |
| SANs/Water | 52.01±1.23 | 1.72±0.02 | 1.78±0.01 |
| TANs/Water | 65.34±0.97 | 1.72±0.02 | 1.69±0.01 |
| S-C-TANs/Water | 67.59±0.78 | 1.77±0.02 | 1.62±0.02 |

**3 The HPLC analysis method for** **andrographolide in plasma samples**

Andrographolide was analyzed at 225 nm using HPLC linked to a Ultimate^Ⓡ^ XB-C18 (4.6 mm ×250 mm, 5μm) column. The mobile phase, consisting of methanol and 0.2% phosphoric acid (46:54, v/v), was pumped at 1 mL/min. The column temperature was 35℃.

**4 gastrointestinal distribution of ADR and different materials5**

The passing times of 50% ADR and polymer materials passed through the fourth (T4(50)), fifth (T5(50)) and sixth (T6(50)) segments of small intestine of SD rats were shown in Table S2, and we can find the distribution of ADR and copolymer (S-C-T) were similar.

**Table S2**

The passing times of 50% ADR and polymer materials passed through the fourth (T4(50)), fifth (T5(50)) and sixth (T6(50)) segments of small intestine of SD rats（mean ± SD, n=3）

| Groups | Time(h) | | |
| --- | --- | --- | --- |
|  | T4(50)/h | T5(50)/h | T6(50)/h |
| SDS | 0.89±0.03 | 1.52±0.12 | 2.40±0.02 |
| TPGS | 0.87±0.06 | 1.67±0.06 | 2.62±0.07 |
| ADR | 0.65±0.02 | 1.16±0.10 | 2.08±0.05 |
| CS | 0.82±0.04 | 1.03±0.14 | 1.30±0.09 |
| SDS-CS-TPGS(S-C-T) | 0.68±0.01 | 0.96±0.01 | 1.61±0.05 |
| S-C-T+ADR/S-C-T | 0.67±0.01 | 0.98±0.06 | 1.04±0.13 |
| S-C-T+ADR/ADR | 0.65±0.04 | 1.02±0.01 | 1.03±0.01 |

**5 Cytotoxicity of nanocrystals**

We explored the toxicity of nanocrystals on MDCK cells, and the results showed these formulations have no cytotoxicity on MDCK cell when the ADR concentration was below 50 μg/mL. When the drug concentration increased to above 100 μg/mL , the toxicity of ADR emerged, and the pure drug nanocrystals (ANs) exhibited lower cytotoxicity than ADR. Because of the cytotoxicity of sodium dodecyl sulfate, S-TANs exhibited certain toxicity, while S-C-TANs has no cytotoxicity (Figure. S1).


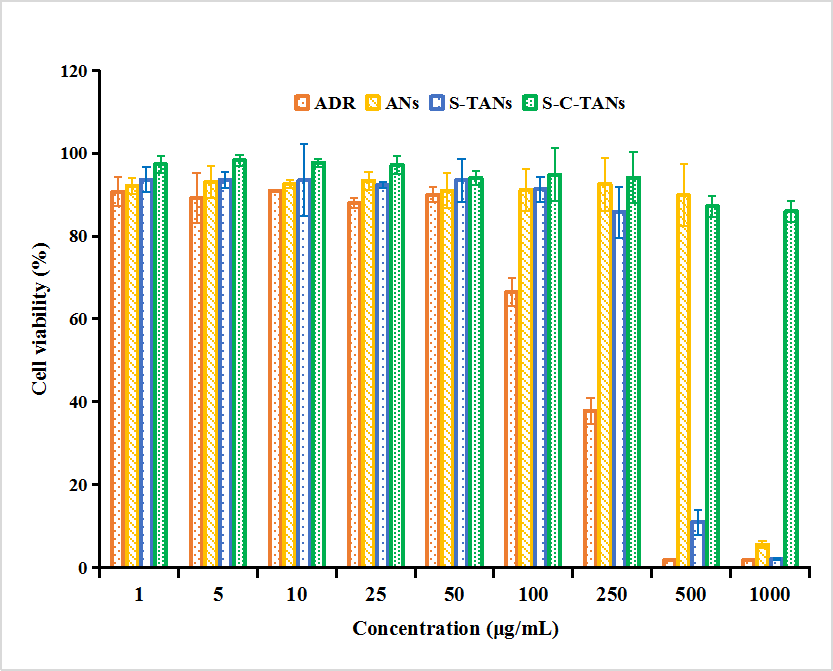


**Figure. S1** Cytotoxicity of different formulations. (mean ± SD, n=3)
